# MetaPro: a web-based metabolomics application for LC-MS data batch inspection and library curation

**DOI:** 10.1007/s11306-023-02018-6

**Published:** 2023-06-08

**Authors:** Shaowei An, Ruimin Wang, Miaoshan Lu, Chao Zhang, Huafen Liu, Jinyin Wang, Cong Xie, Changbin Yu

**Affiliations:** 1 Fudan University, 220 Handan Road, Shanghai, 200433 China; 2 Westlake University, 18 Shilongshan Road, Hangzhou, Zhejiang Province 310024 China; 3 Shandong First Medical University, 6699 Qingdao Road, Jinan, Shandong Province 250117 China; 4 Calibra Diagnostics Co., Ltd, 329 Jinpeng Street, Hangzhou, Zhejiang Province 310030 China; 5 Carbon Silicon (Hangzhou) Biotechnology Co., Ltd, 368 Jinpeng Street, Hangzhou, Zhejiang Province 310030 China; 6 Zhejiang University, 866 Yuhangtang Road, Hangzhou, Zhejiang Province 310009 China

**Keywords:** Metabolomics, Software, Mass spectrometry, Workflow, Database, Spectral library, Quality control, Quality assurance, Metabolite identification, Web-based

## Abstract

**Introduction:**

Metabolomics analysis based on liquid chromatography-mass spectrometry (LC-MS) has been a prevalent method in the metabolic field. However, accurately quantifying all the metabolites in large metabolomics sample cohorts is challenging. The analysis efficiency is restricted by the abilities of software in many labs, and the lack of spectra for some metabolites also hinders metabolite identification.

**Objectives:**

Develop software that performs semi-targeted metabolomics analysis with an optimized workflow to improve quantification accuracy. The software also supports web-based technologies and increases laboratory analysis efficiency. A spectral curation function is provided to promote the prosperity of homemade MS/MS spectral libraries in the metabolomics community.

**Methods:**

MetaPro is developed based on an industrial-grade web framework and a computation-oriented MS data format to improve analysis efficiency. Algorithms from mainstream metabolomics software are integrated and optimized for more accurate quantification results. A semi-targeted analysis workflow is designed based on the concept of combining artificial judgment and algorithm inference.

**Results:**

MetaPro supports semi-targeted analysis workflow and functions for fast QC inspection and self-made spectral library curation with easy-to-use interfaces. With curated authentic or high-quality spectra, it can improve identification accuracy using different peak identification strategies. It demonstrates practical value in analyzing large amounts of metabolomics samples.

**Conclusion:**

We offer MetaPro as a web-based application characterized by fast batch QC inspection and credible spectral curation towards high-throughput metabolomics data. It aims to resolve the analysis difficulty in semi-targeted metabolomics.

**Supplementary Information:**

The online version contains supplementary material available at 10.1007/s11306-023-02018-6.

## Introduction

Metabolomics, or metabolite profiling, has become a powerful approach to providing a functional readout of cellular biochemistry (Patti et al., 2012). Currently, one of the most momentous metabolomics analysis platforms is liquid chromatographic separation techniques coupled to accurate tandem mass spectrometry (LC-MS/MS) as it allows the physical separation of thousands of metabolites, thus providing a more comprehensive view of the metabolome (Blaženović et al., 2018). During the metabolomics analysis process, one experimental sample could generate thousands of metabolite signals. However, accurately quantifying these signals remains a great challenge in the metabolomics field due to the intrinsic complexity of LC-MS data.

The challenges of accurate metabolomics analysis arise from several aspects. First, the reproducibility crisis in metabolomics and the lack of transparency in quality assurance (QA) and quality control (QC) might lead to ambiguity among labs(Kirwan et al., 2022). Many factors, such as sample handling, pre-processing, and measurement, could result in variations in analysis outcomes. Reducing these impacts by methodology is of great concern for many software developers. Although many pieces of metabolomics software like NOREVA (Li et al., 2017) have been developed to minimize these variations, manual inspection is still indispensable in many analysis processes. Specifically, quantifying metabolite peaks is difficult to verify when facing hundreds of experimental samples. Additionally, many pieces of metabolomics software directly output the quantification results without offering manual modification functions toward the quantification results. This results in the circumstance that many labs lack the conditions or instructions to conduct normative analysis (Dunn et al., 2017).

Several methods have been developed for identifying metabolites (Nguyen et al., 2019; Wishart, 2009), but the most prevalent strategy is comparing a given spectrum with curated spectral databases such as HMDB (Wishart et al., 2022) and METLIN (Smith et al., 2005). However, the metabolite identification accuracy of this method is highly dependent on the quality of the spectral library. A reliable spectral library can increase the confidence level and accuracy of analysis results. Unfortunately, there is always a lack of spectra of many metabolites in public or commercial spectral libraries. Encouraging the creation of homemade spectral libraries can help address this problem and enable the metabolomics community to accumulate and document more high-quality spectra of unknown metabolites.

As mentioned above, there is an urgent need to develop a platform that can resolve analysis difficulties and spectra shortages. In addition, computation optimization and industrial-grade framework development applied in software can increase laboratory efficiency in handling huge amounts of metabolomics data. Here we present MetaPro, a web-based metabolomics analysis application characterized by user-friendly batch QC inspection and reliable library curation towards high-throughput LC-MS semi-targeted (Viant et al., 2019) data analysis. Additionally, MetaPro offers easy-to-use interfaces to help users minimize analysis bias among samples and improve quantification accuracy by optimized peak detection algorithms. Analysis of the case study demonstrates the applicability of MetaPro in the metabolomics analysis workflow and how it improves identification accuracy by reusing collected authentic or high-quality spectra. Using industrial-grade development techniques, MetaPro is able to analyze tens of thousands of LC-MS data samples daily. Its web-based application characteristics make simultaneous analysis especially superior on this platform. We expect MetaPro to reinforce the concepts of QC in the metabolomics community, improve laboratory analysis efficiency, and offer the spectra curation function for researchers.

## Materials and methods

### Analysis acceleration by the computation-oriented data format

The MetaPro application is accelerated by using a computation-oriented data format named Aird (Lu et al., 2022), which is characterized by a higher compression ratio and less decoding time. Aird can support more efficient file reading due to its computation-oriented data arrangement and index structure. Before analyzing on MetaPro, all raw files from different mass spectrometry instruments should be converted to Aird files. Owing to the usage of this data format, the storage space of experimental files is reduced to about 50% of its original size, and the computation speed is accelerated as well. The process to convert raw files to Aird files is described in the user manual.

### Implementation

**Fig. 1:**
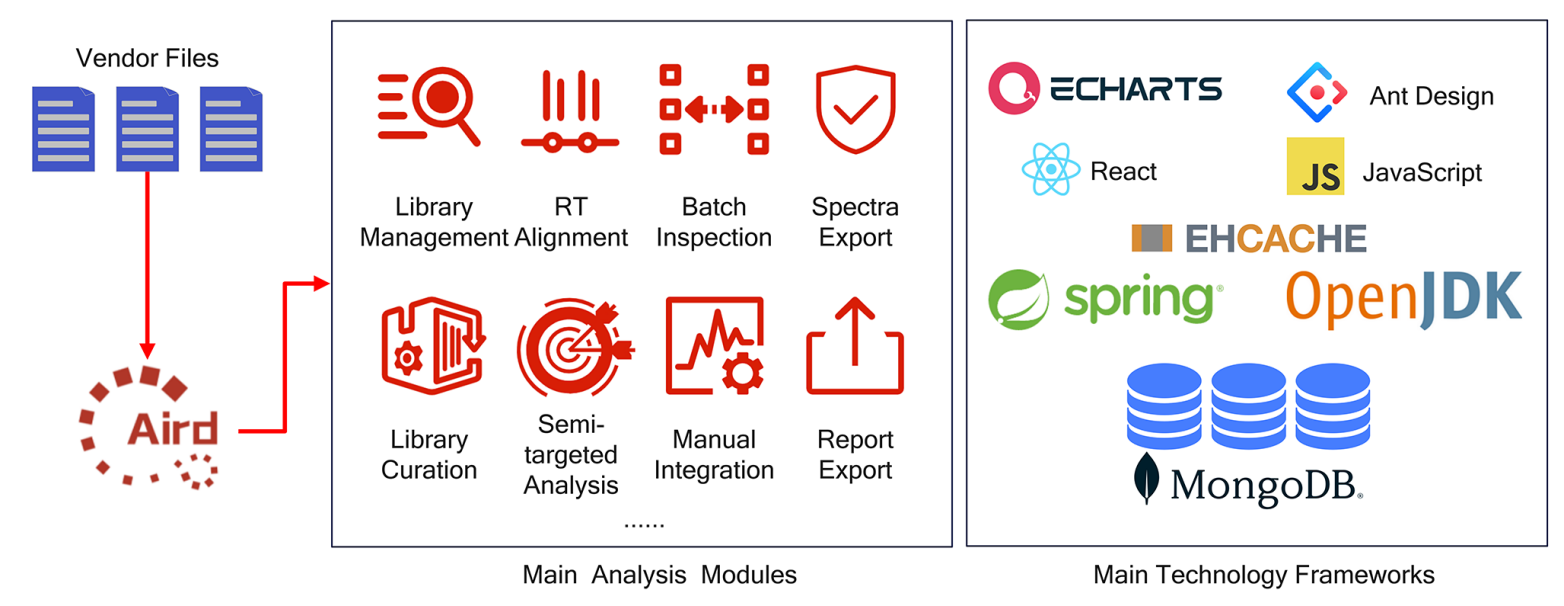
MetaPro analysis modules and implementation framework. MetaPro uses the Aird data format for acceleration in computation speed and thus all the vendor files need to be converted to Aird files, which are regarded as the input for MetaPro. The main analysis modules on MetaPro include library management, library curation, RT alignment, semi-target analysis, batch inspection, manual integration, spectra export, and report export. The main technology frameworks appearing in the figure were adopted for the front-end, back-end, and database in the development stage

The whole implementation framework is shown in Fig. 1. MetaPro is an application designed under the production-grade framework Spring Boot and has separate front-end and back-end technologies. The front-end framework is written in JavaScript while the back-end framework is developed in Java. To achieve highly dynamic and interactive visualization effects, Echarts (Li et al., 2018a), React, and Ant Design are used collaboratively on the front end of MetaPro to support various visualization effects. MetaPro has a well-defined document-based data storage structure and adopts MongoDB for reliable data management.

### Requirements

MetaPro is available for Windows, Linux, and MacOS. The minimum and recommended system installation requirements are listed in Table S1. All the frameworks contained in the program are packaged into a zip file. MetaPro supports “one-click” type installation. Currently, MetaPro only supports MS data analysis acquired using data-dependent acquisition (DDA).

### Automatic and flexible quantification

MetaPro performs quantification through eight steps, which include extraction of extracted ion chromatograms (EIC), EIC smoothing, EIC noise estimation, peak selection, peak integration, peak screening, peak identification, and batch optimization. In EIC extraction, MetaPro uses fast extraction algorithms to achieve rapid EIC extraction from Aird files. MetaPro has undertaken long-term data-driven optimization and iteration of the analysis methods from mainstream metabolomics and proteomics software, such as XCMS (Smith et al., 2006), MS-DIAL (Tsugawa et al., 2015) and MZmine3(Schmid et al., 2023). MetaPro provides a variety of data analysis methods to meet the analysis requirements of different users on different datasets, including four EIC smoothing methods, three EIC noise estimation methods, three peak selection methods, two peak integration methods, five peak screening criteria, and three peak identification methods. The explanation and recommended values for each method or parameter can be found in Table S2. In batch analysis scenarios, MetaPro improves the efficiency of manual inspection through automated result optimization algorithms, including a compound-by-compound multi-file EIC alignment algorithm to ensure peak selection consistency, and a pattern recognition algorithm to improve the accuracy of peak selection based on RT distributions of adjacent compounds in the library. Diagrams of how these algorithms work are exhibited in Fig S1.

## Results

### Design conception

MetaPro aims to provide users with batch QC inspection for accurate quantification results and homemade spectra library construction functionality. We note that in the metabolomics data analysis process, there remain difficulties in thoroughly avoiding manual inspection. Thus, artificial judgment and algorithm inference can be combined to achieve more accurate outcomes. Based on this concept, we take MetaPro as a useful tool for aiding metabolomics experts to improve their working efficiency through fast inspection and accurate recommendations.

According to MSI (Sumner et al., 2007; Tada et al., 2019) standards, the most reliable identification (level-1) requires at least two orthogonal properties matching between a compound and its authentic standard analyzed under identical experimental conditions. Normally accurate mass and retention time (RT) are considered to be the matching criteria in LC-MS analysis. However, owing to the co-eluting phenomenon and RT fluctuations of certain chromatography techniques, this criterion might not be sufficient to reliably identify compounds (Tada et al., 2019). Furthermore, “literature values reported for authentic samples by other laboratories are generally believed to be insufficient to validate a confident and rigorous identification”(Sumner et al., 2007). For this reason, MetaPro encourages users to identify and quantify a metabolite according to a homemade database with four matching standards: accurate mass, RT, MS1 spectrum, and MS2 spectrum.

From the software development perspective, how metabolomics data are organized in an application is of great concern to us. MetaPro consists of these basic metabolomics study elements: compound, experiment, library, method, overview, project, spectra, and task. The implication for each element is illustrated in Table S3.

### Workflow

**Fig. 2:**
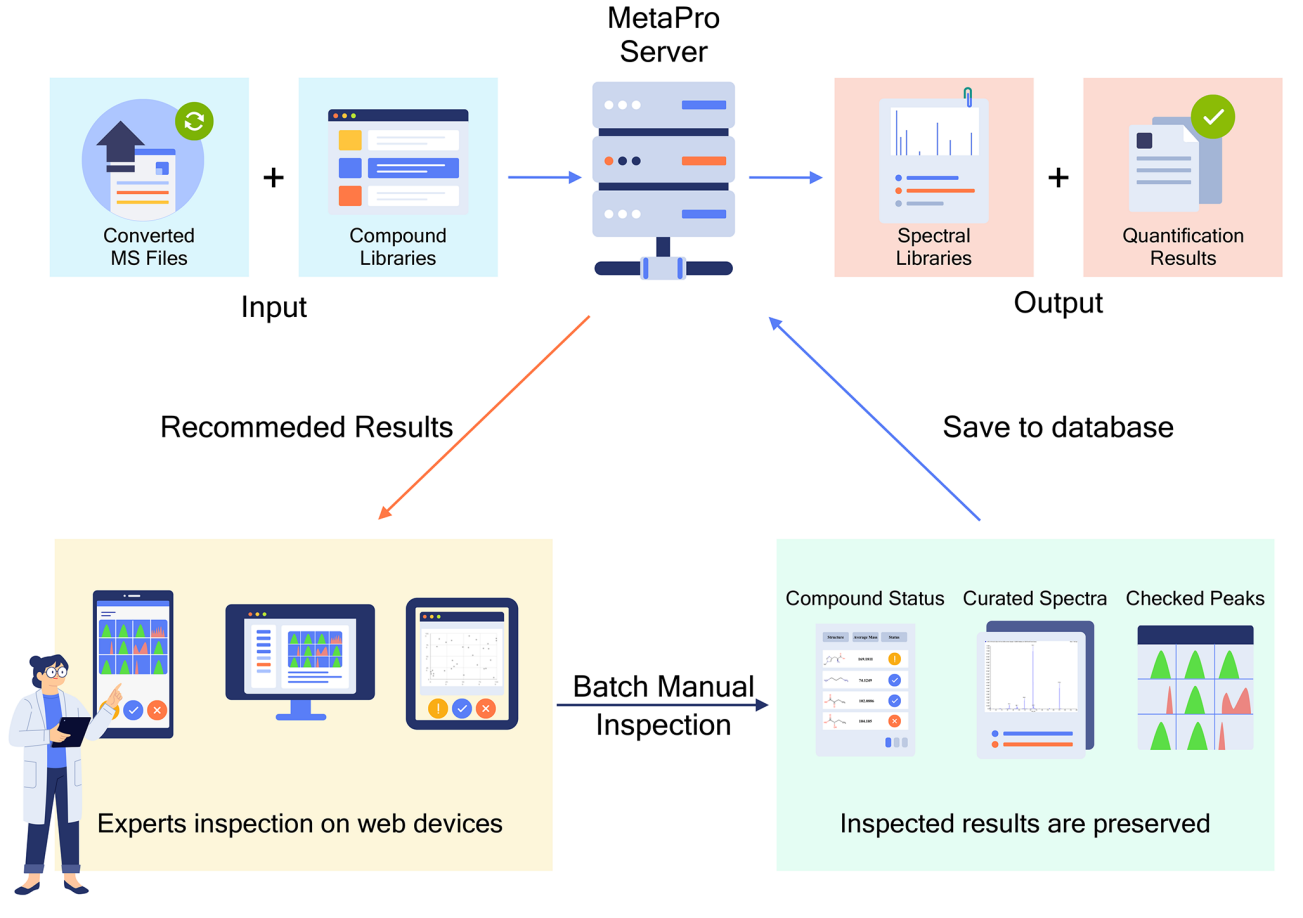
The main workflow of MetaPro. The converted MS files (Aird files) and the compound libraries should be prepared as the input for MetaPro. After the automatic calculation process is complete, users can browse algorithm-recommended results through various web browsers. Batch inspection operations help users view peak shapes and spectra in highly reactive and efficient interfaces. Inspected compounds, peaks, and spectra can be selected for saving to the database and will be used for the next analysis cycle

The main workflow of MetaPro is shown in Fig. 2. First, users need to convert their vendor files from different instruments to Aird files using the AirdPro client. Then, they input compound libraries with accurate m/z and RT into MetaPro. MetaPro executes extraction analysis on these Aird files and recommends peaks and related spectra to the users for further manual inspection. After the manual inspection of these results is finished, users can export quantification results or approved spectra. Approved spectra can also be saved into the database for metabolite identification in other projects.

This workflow helps accumulate inspected spectra into the database and improves identification accuracy through circulation if users have collected adequate authentic or high-quality spectra. Specifically, once users search for a specific compound in their samples, they check the peak shape and consider reserving some high-quality MS/MS spectra from these samples. The check process has different meanings when facing different situations. For metabolites with known spectra, it means comparison with known databases and ensuring the quality of users’ curated spectra. For metabolites without known spectra, it means users can reserve these spectra temporally and observe their consistency in other experimental files. If some spectra demonstrate long-term consistency in users’ experimental files, they could be considered for further identification.

After users’ spectral library reaches a certain scale, our algorithms allow them to compare the spectra in a new sample and their former recorded spectra according to a combination of seven scores (Table S2), and each score weight can be user-defined. Through this workflow circulation, not only is the spectral library on their local computer extended but also the identification accuracy is improved if based on an enlarged high-quality reference database.

### Semi-targeted analysis function

The semi-targeted analysis function requires pre-prepared lists of compounds of interest that will be used for chromatogram extraction and peak picking. The prepared compound lists are named internal standard (IS) libraries or analyte libraries in the system. Users can choose whether they want to use the IS library for RT calibration. If both the IS library and analyte library are provided to MetaPro, the RT in the analyte library will be adjusted according to the RT in the IS library.

After the library is prepared, starting semi-targeted analysis on a project will let MetaPro automatically extract the chromatograms and save recommended peak detection results into the database for further inspection and modification. The algorithms and methods used in this step are described in the former section.

The semi-targeted analysis outcomes on a certain batch are saved as an overview every time. These overviews are browsable and manageable for users on MetaPro. Overviews also record a series of parameters used in the analysis process for convenient future queries. After the results are generated, users can proceed to the next step to check these results in batches.

### QC and batch inspection interface

**Fig. 3:**
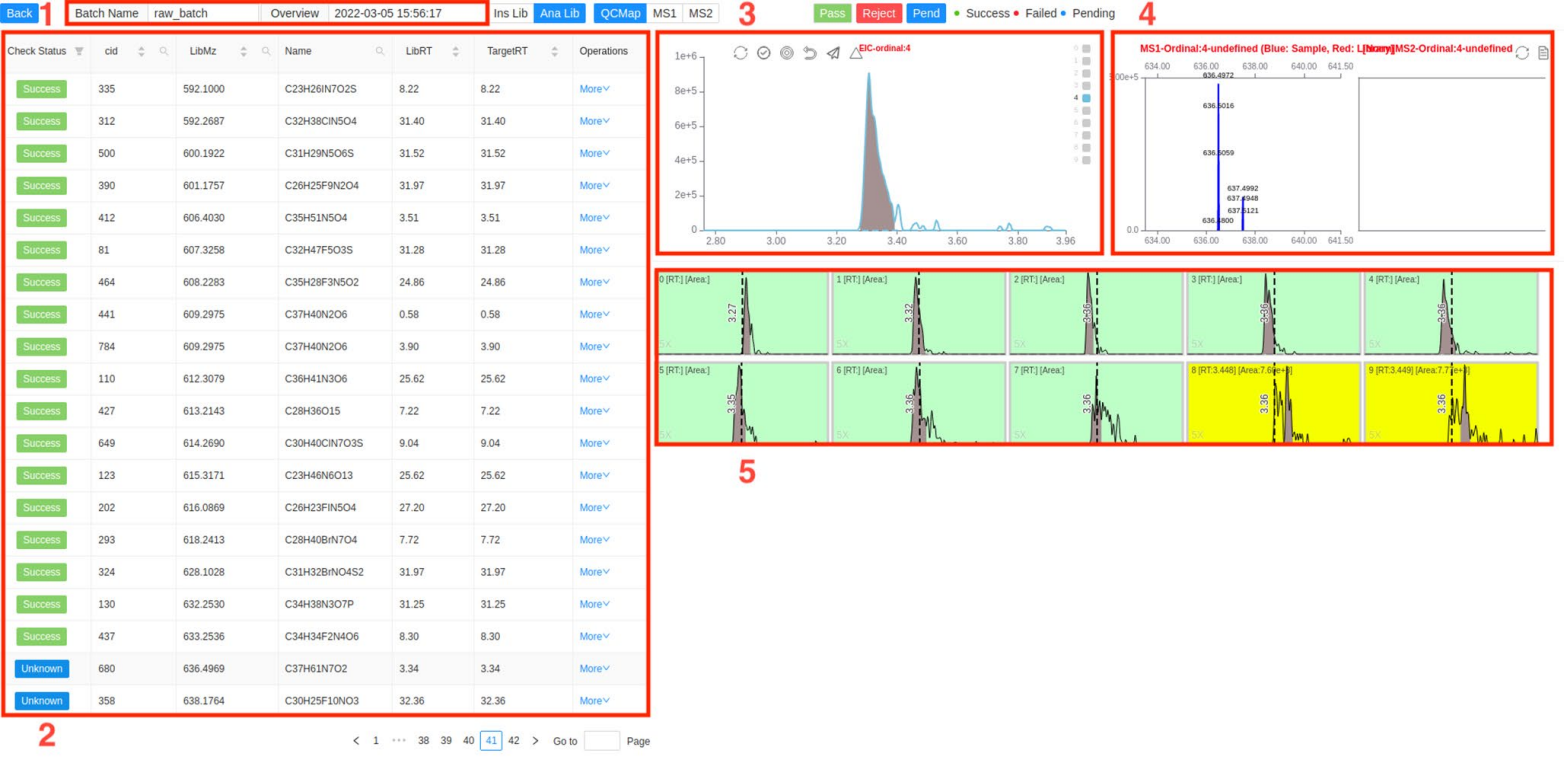
The layout of the batch inspection interface. (**1**) The overview switch module. (**2**) The compound switch module. (**3**) The manual integration module. (**4**) The spectrum viewer module. (**5**) The peak shape batch inspection module

The batch inspection interface mainly consists of the following modules. The layout of this interface is shown in Fig. 3.

#### Overview switch module

This module lies at the top of the page and controls the current overview. All the overviews are labeled with time so users can switch to a specific overview quickly according to the recorded time.

#### Compound switch module

On the left of the page is the compound controller module, allowing users to quickly switch the current compound of interest in the library. Selecting a compound here will focus the whole page on this specific compound among all the samples. Users can also change the expected RT and peak identification parameters of a certain compound and apply them to all the samples.

#### Peak shape batch inspection module

This module displays all the peaks selected for a certain compound among all the samples in a batch. Moving the pointer to a peak allows users to check the recorded peak information. Different background colors (green, yellow, red) represent different check statuses (pass, unknown, fail) for samples. Users can change the status of a sample to annotate its manual check outcome. If a sample peak is labeled red or yellow, it will not show up in the final result report. Choosing multiple samples can check for peak overlap between these samples.

#### Manual integration module

If users find that the algorithm’s peak picking result in a sample is not up to their expectation or QC standard, they can double-click on the sample to manually modify the peak in this module. The fast manual inspection would help revise wrongly chosen peaks.

#### Spectrum viewer module

This module contains two graphical charts displaying the MS spectrum and the corresponding MS/MS spectrum. Users could check the peak apex spectrum and neighbor spectra of a peak by clicking on the corresponding positions, which helps them check all the MSI needed information.

In addition to these functional modules, keyboard shortcuts are optimized specially for quick inspection and status annotation. When the system is deployed on a high-performance computing server, it can support multiple users collaborating on the same project to inspect results. This interface would help researchers work efficiently and in perfect order when they require metabolomics analysis on a large scale of samples, which might refer to thousands of metabolites on thousands of samples. In realistic application scenarios, one skilled user on MetaPro can process 50 experimental files with a library containing 1000 compounds (500,000 peaks to be inspected) within 8 h.

### Spectra library curation

Here we introduce the spectra library curation and management system. The main aim of this function module is to encourage the construction of homemade spectra libraries. By accumulating spectra from users’ samples, frequently appearing spectra in users’ labs or spectra appearing under certain circumstances could be used for further metabolite identification after a manual check. Expanding the spectral library through iteration would accumulate metabolite references and benefit the metabolomics research community.

The prerequisite for conducting spectra library curation is an analyzed and carefully inspected result. Users need to choose the checked overviews to start the library curation process. After the library curation process begins, the system will automatically save approved spectra from the project. Spectra approved by users are gathered according to the metabolites they belong to. At the same time, spectra information such as instrument type, collision energy, ionization mode, and file source is recorded into the database as well.

Users can manage collected spectral libraries with rich visualization and modification functions that aim to avoid selecting low-quality spectra. Operations like multi-selection or multi-deletion are equipped with easy-to-use shortcuts on this page.

## Case study

To illustrate how this application is used for metabolomics data analysis, we chose two public benchmark datasets (Z. Li et al., 2018b) to analyze on MetaPro. In these datasets, 1100 compounds were separated into seven groups (Gd1, Gd2, Gd3, Gm, Gd4, Gd5, Gd6) and mixed into two standard mixtures SA and SB. The group Gm has 970 compounds with an equal concentration in SA and SB, while the other 130 compounds have various concentration ratios of 1/16, 1/4, 1/2, 2/1, 4/1, and 16/1 in SB: SA respectively. The standard mixtures were analyzed by two HRMS platforms, AB SCIEX TripleTOF6600 interfaced with Shimazu L30A UPLC and Thermo Q Exactive HF with Dionex UltiMate 3000 HPLC.

**Fig. 4:**
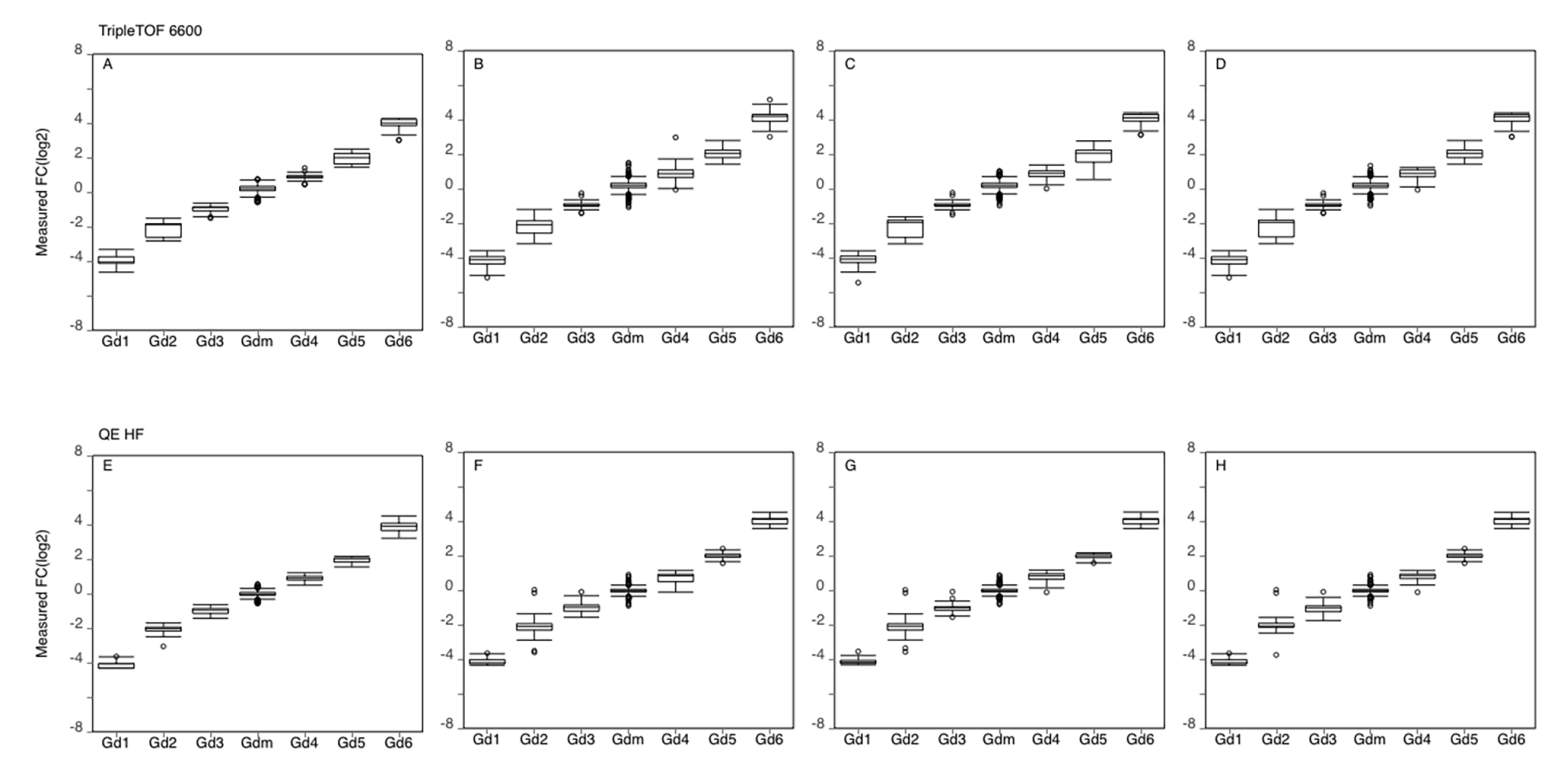
Relative quantification results for compounds in the benchmark datasets. All the results are presented as log-transformed fold changes of compounds in SB: SA. The manually verified results from the software provided by the instrument manufacturers in the TripleTOF 6600 dataset (**A**) and QE HF dataset (**E**). The results measured by MetaPro in the first (**B**), second (**C**), and third run (**D**) in the TripleTOF 6600 dataset and in the first (**F**), second (**G**), and third run (**H**) in the QE HF dataset

We ran each dataset on MetaPro three times with the same parameter settings. In the first run, we performed semi-targeted quantification with a compound library that contains no spectra and exported the raw quantification results without any manual inspection. In the second run, we manually inspected all the quantification results with the batch inspection function on MetaPro and exported them. In the third run, we first collected the manually inspected spectra from the second run and then conducted an analysis based on the curated library. The results are shown in Fig. 4.

The figure displays log-transformed relative quantification results under different analytical conditions. MetaPro demonstrated its quantification accuracy and stability in the comparison of the result distribution before and after manual inspection. In addition, MetaPro achieved better quantification results by analyzing with a built spectral library compared to the result distribution without the spectral library, which proved the effectiveness of self-made library curation. This forms a mutually beneficial relationship where manually inspected results bring accurate spectral libraries, and accurate spectral libraries promote more accurate data analysis, resulting in a more efficient manual inspection.

Benefiting from the accurate analysis algorithm and the user-friendly batch review function, we performed an extremely efficient manual inspection in this study. One person manually inspected 16,120 peaks in 18 files within three hours. A detailed experimental running record of this case study could be found in Table S4. Besides, a supplementary experiment to this case study was conducted and the results could be seen in Fig S2.

## Conclusion and discussion

We introduce MetaPro as a web-based application that offers fast batch inspection and library curation features for high-throughput metabolomics data. The application was designed with a focus on effective quality control and combines algorithm optimization with artificial judgment. The iterative amplification of homemade spectra improves the potential identification accuracy on this platform. The industrial-grade development framework makes the application applicable to many user scenarios. MetaPro has been applied to some biotechnology companies and proved its usability on a large number of real experimental samples. Equipped with many interactive graphical interfaces and useful functions, MetaPro enhances the efficiency of metabolomics analysis results review.

What we want to discuss here is the future direction of MetaPro’s evolution. For many researchers, untargeted analysis plays a crucial role in their investigations. Currently, MetaPro only addresses the analysis difficulty in known mass and RT compounds. Unknown feature identification from users’ samples needs to be done using software with related functions. The untargeted analysis workflow and unknown metabolite identification functions are under development and will be supported in the next release. This will make MetaPro a one-stop application applicable for most kinds of MS-based metabolomics analysis.

## Electronic supplementary material

Below is the link to the electronic supplementary material.

Supplementary Material 1

Supplementary Material 2

## Data Availability

The software is freely available at https://github.com/CSi-Studio/MetaPro. Vendor files, Aird files, and compound lists for the case study can be found at https://zenodo.org/deposit/6326825.
