# Supplementary material for: MetaPro: a web-based metabolomics application for LC-MS data batch inspection and library curation
Source: Metabolomics. 2023 Jun 8;19(6):57. doi: 10.1007/s11306-023-02018-6 (PMC10250499; doi:10.1007/s11306-023-02018-6)
Supplement: Supplementary file 1 — Supplementary Material 1 [file 11306_2023_2018_MOESM1_ESM.docx]

**SUPPLEMENTARY DATA**

Fig S1. Main algorithm processes in MetaPro.


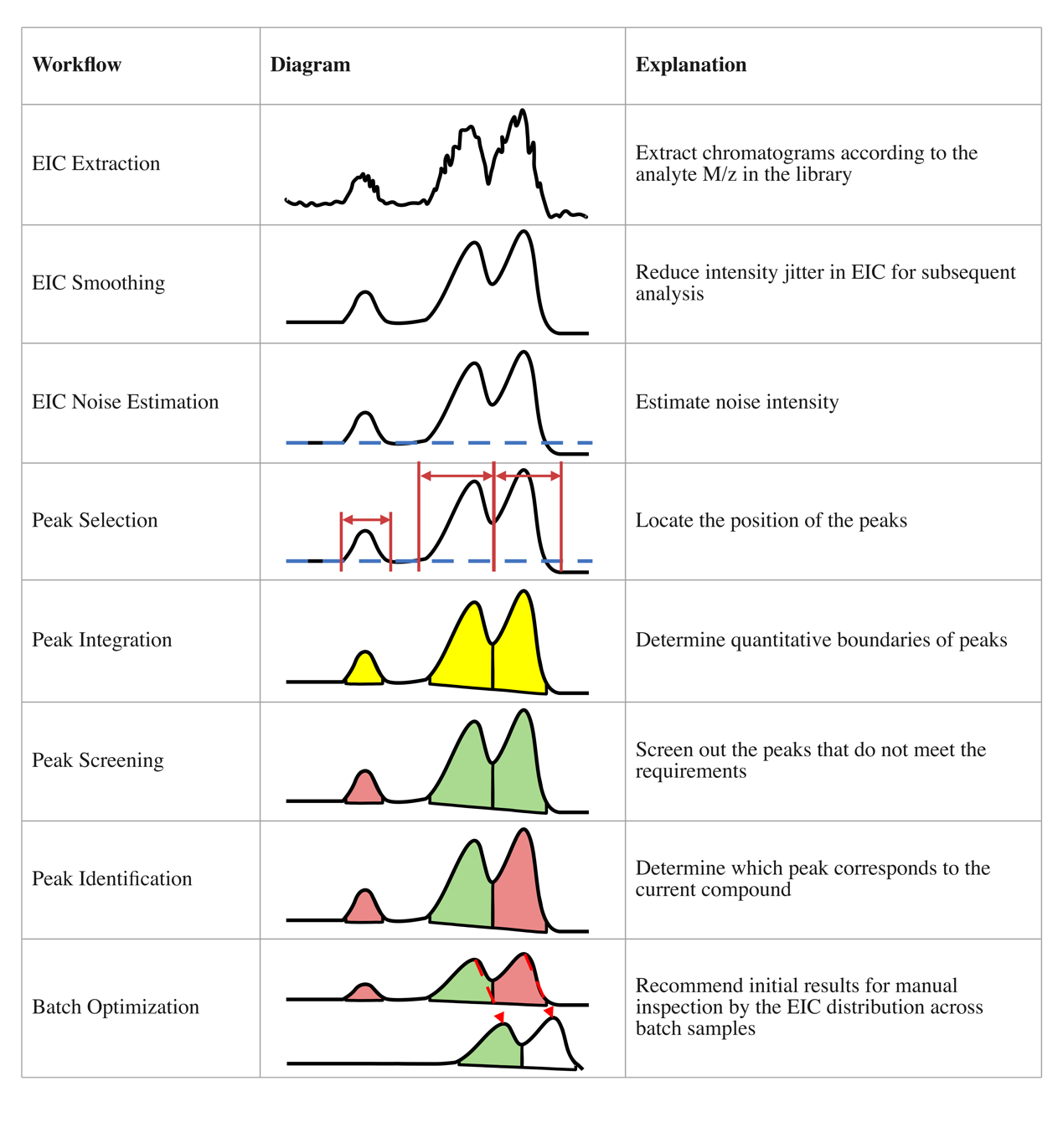


Fig S2. Relative quantification deviation averages and CV values of four analysis strategies towards two reference standards in the two datasets.


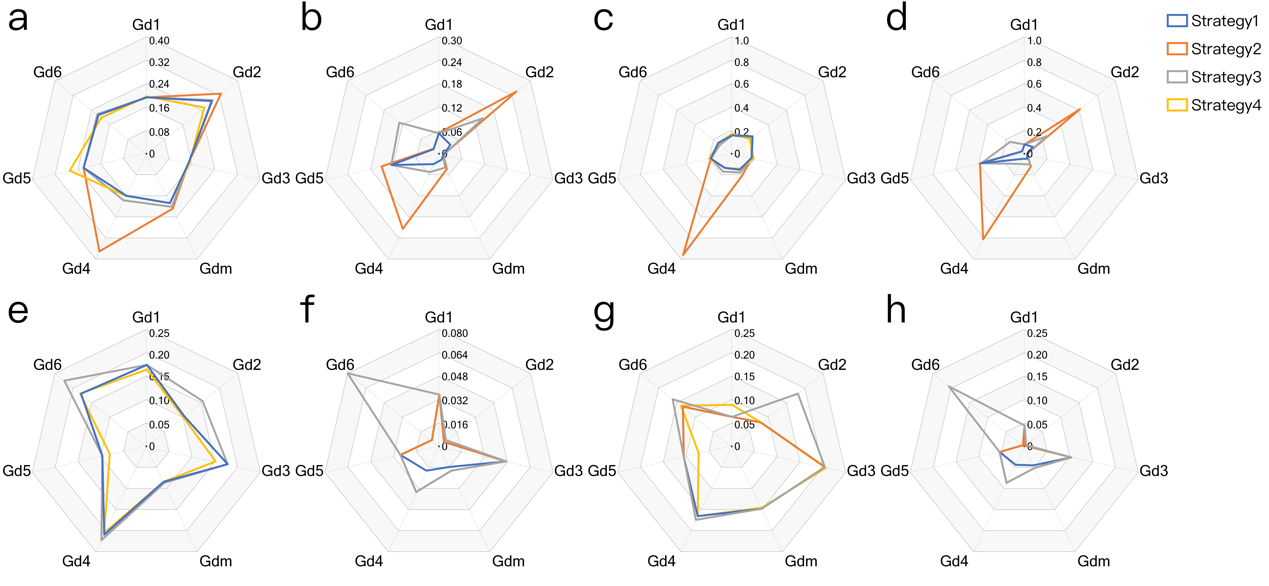


(a, b) The averages towards Standard 1 and Standard 2 in the TripleTOF 6600 dataset. (c, d) The CV values towards Standard 1 and Standard 2 in the TripleTOF 6600 dataset. (e, f) The averages towards Standard 1 and Standard 2 in the QE HF dataset. (g, h) The CV values towards Standard 1 and Standard 2 in the QE HF dataset.

**Strategy 1:** Analyze using the Highest Score peak identification method with a constructed high-quality spectral library.

**Strategy 2:** Analyze using the Nearest RT method without a constructed spectral library.

**Strategy 3:** Analyze using the Highest Score peak identification method without a constructed spectral library.

**Strategy 4:** Analyze using the Highest Score method and go through the manual inspection and modification.

**Standard 1:** The theoretical compound ratios from the experimental design.

**Standard 2:** The relative quantification ratios for SB: SA in each group from Strategy 4.

This figure supplements Figure 4 and reveals similar conclusions. Firstly, a curated high-quality spectral library would help improve quantification accuracy. This is evident from the comparison of Strategy 1 and Strategy 3. The result from Strategy 1 has smaller deviation values and CV values in most groups compared to Strategy 3. Using a well-constructed spectral library reduced the average deviation by **52.2% (18.5%)** and the CV values by **48.5% (16.2%)** among all metabolites in the TripleTOF 6600 dataset (QE HF dataset) according to Standard 2. This hints that reference to the manually checked spectral library helps the algorithm find peaks more accurately.

Secondly, the combination of the seven peak scores (see Table S2) is better than merely referencing theoretical RT to identify a peak, as seen by the comparison of Strategy 2 and Strategy 3. Theoretical RT takes a crucial position to find a peak but might not be sufficient.

Thirdly, the quantification results from Strategy 1 were very similar to those from Strategy 4. This shows that quantification accuracy on MetaPro is very close to manual inspection results if using a well-constructed spectral library to conduct analysis. This system could thus improve experts’ efficiency when dealing with the QC process.

Overall, these results validate the analysis process on MetaPro through various comparisons.

Table S1. System requirements.

| Specifications | Minimum | Recommendation |
| --- | --- | --- |
| CPU | Intel Core i5 10-gen or AMD R5 | Intel Core i9 12-gen or AMD R9 5900 |
| Hard drive | 50GB free space | 2x dataset size |
| RAM | 16GB | 64GB(Core i9-10Gen),  128GB(Core i9-12Gen) |

Table S3. System item explanation.

| Item | Explanation |
| --- | --- |
| Compound | A compound refers to a specific metabolite whose name, ID, m/z, RT and other information will be stored in the database. Selected compounds could be used for conducting targeted analysis. |
| Experiment | An experiment is a basic analytical unit in the MetaPro. It represents the mass spectrometry data obtained from one sample. The actual storage mode of an experiment is an Aird format file under the user-defined document folder. A few experiments can be divided into one batch. |
| Library | A library contains a few compounds. |
| Method | All parameters used in the analysis steps are extracted into a method. Different parameters composition could be saved into different methods for convenient query. |
| Overview | An overview is the storage of the analyzed results. By checking a overview, multiple quality control operations can be achieved. |
| Project | A project can have a few batches. Each batch has a few experiments. Project is the largest unit where experiments could be organized. |
| Spectrum | A spectrum is correlated to a specific compound. Instrument type, collision energy, ionization mode and other description information of the mass spectrometry characteristics are stored in a spectrum. |
| Task | Task refers to a metabolomics analysis process needed to be done or undertaken. It’s timely status and records could be seen in the task page. |

Table S4. Case study running record.

| Parameters | Values |
| --- | --- |
| Computer | MacbookPro 16 (2019) |
| CPU | 2.3GHz 8 core i9 |
| Memory | 32GB |
| OS | macOS Monterey 12.5 |
| Time cost for Thermo QE HF samples | 44458(ms) |
| Time cost for TOF6600 samples | 32310(ms) |
| Timm cost for manual inspection | Almost 3 hours |
